# Adrenergic Blockade by Nebivolol to Suppress Oral Squamous Cell Carcinoma Growth *via* Endoplasmic Reticulum Stress and Mitochondria Dysfunction

**DOI:** 10.3389/fphar.2021.691998

**Published:** 2021-08-12

**Authors:** Qian Chen, Han Jiang, Zhen Wang, Lu-Yao Cai, Yu-Chen Jiang, Liang Xie, Yu Zhou, Xin Zeng, Ning Ji, Ying-Qiang Shen, Qian-Ming Chen

**Affiliations:** State Key Laboratory of Oral Diseases, National Clinical Research Center for Oral Diseases, Chinese Academy of Medical Sciences Research Unit of Oral Carcinogenesis and Management, West China Hospital of Stomatology, Sichuan University, Chengdu, China

**Keywords:** nebivolol, oral squamous cell carcinoma cells, endoplasmic reticulum stress, mitochondria, autonomic nerve

## Abstract

Adrenergic nerve fibers in the tumor microenvironment promote tumor growth and represent a potential target for cancer therapy. However, the effectiveness of targeting adrenergic nerve fibers for oral squamous cell carcinoma (OSCC) therapy needs to be evaluated by preclinical data. Herein, the 4NQO-induced and orthotopic xenograft OSCC mice models were established. We demonstrated that using 6OHDA chemical denervation as well as using nebivolol adrenergic blockade could halt the oral mucosa carcinogenesis. Our preclinical studies suggested that nebivolol, which is widely used to treat cardiovascular diseases, can be repositioned as a potential candidate to treat OSCC. Remarkably, we revealed the precise effect and mechanism of nebivolol on OSCC cells proliferation, cell cycle, and cell death. Administration of nebivolol could activate the endoplasmic reticulum (ER) stress signaling pathway through increasing the expression of inducible nitric oxide synthase, which subsequently triggers the integrated stress response and cell growth arrest. Simultaneously, ER stress also induced mitochondrial dysfunction in OSCC cells. We found that the accumulation of dysfunctional mitochondria with the impaired electron transport chain caused increasing reactive oxygen species production, which ultimately resulted in OSCC cell death. Altogether, our finding suggested a novel therapeutic opportunity for OSCC by targeting adrenergic nerve fibers, and repurposing nebivolol to treat OSCC can be represented as an effective strategy.

## Introduction

Oral squamous cell carcinoma (OSCC) is the most common head and neck malignant tumor, which is characterized by poor prognosis, high recurrence rates, and difficult treatment (Erratum, 2020). Currently, the major therapeutic strategy for OSCC cells is surgery plus radiotherapy or chemotherapy (Zhang et al., 2020). However, these widely approved treatments may bring severe adverse and drug resistance. Therefore, developing effective therapeutics for OSCC may provide the key to improve the survival rate of OSCC patients.

The component from the tumor microenvironment (TME) may serve as a potential drug target in the treatment of cancer. TME is the internal environment for tumor occurrence and development, which is constituted with cells and extracellular matrix, including tumor cells, stromal cells, blood vessels, lymphatic vessels, nerves, and other tissue structures (Hui and Chen, 2015; Hinshaw and Shevde, 2019; Vitale et al., 2019). Inside the TME, nerves are emerging as regulators to affect cancer initiation, progression, and metastasis. Some pioneer studies have shown that autonomic nerve fibers could regulate tumor progression by secreting neurotransmitters, which could bind with the corresponding receptors on the surface of tumor cell membranes (Zahalka and Frenette, 2020). The newly discovered role of nerves in regulating cancer progression offers an opportunity to develop therapeutic strategies. Alternatively, pharmacological blockade of neurotransmitter receptors might be a more suitable therapeutic strategy. The peripheral adrenergic nervous system has previously been shown to accompany the initial phase of OSCC development and promote OSCC growth. Hence, adrenergic stimulation provides opportunities for repurposing β-blockers to OSCC therapy.

Nebivolol, the third-generation β-blocker with vasodilator function, has been approved by the Food and Drug Administration (FDA) for the treatment of cardiocerebrovascular diseases almost for 5 years (Cicero et al., 2018). Nebivolol could enhance nitric oxide (NO) production in endothelial cells and biased agonism. Additionally, nebivolol displayed antioxidant activity *via* direct free radical scavenging and inhibition of NADPH oxidase activity (Bhadri et al., 2018). A recent study also demonstrated that nebivolol inhibited complex I and ATP synthase activities and arrested angiogenesis to halt colon and breast tumor growth (Nuevo-Tapioles et al., 2020). In light of these findings, adrenergic blockade by nebivolol seemed to be an attractive approach for OSCC treatment and the effectiveness of nebivolol for OSCC therapy needs to be evaluated by preclinical data.

Herein, we investigated the distribution of the nerves in OSCC and explored the effect of denervation-based cancer therapies for OSCC by using 6OHDA. We also tested the possibility to halt OSCC progression by nebivolol administration. Besides, we studied the underlying mechanism by an adrenergic blockade by nebivolol-exerted cytotoxicity in OSCC. Therefore, it is speculated that nebivolol seems to be a candidate therapeutic drug for the treatment of OSCC.

## Materials and Methods

### Cell Lines and Reagents

OSCC cell lines (HSC-3 and HN12) were obtained from the Japanese Collection of Research Bioresources (JCRB) Cell Bank. The cells were routinely cultured in high glucose DMEM supplemented with 10% fetal bovine serum (Invitrogen Life Technologies, Carlsbad, CA, United States) and 1% antibiotics at 37°C in a 5% CO_2_ incubator. The primary antibodies anti-PERK, anti-p-PERK, anti-eIF2α, anti-p-eIF2α were purchased from Cell Signaling Technology (1:1000, United States). The primary antibodies anti-ATF4, anti-caspase-3, anti-cleaved-caspase-3, anti-Bcl2, anti-Bax were obtained from Affinity (1:500, United States). Antibodies α-tubulin, anti-HSP60, anti-IF1, anti-VDAC, and anti-CHOP were obtained from Abcam (1:1000, Cambridge, MA). The OXPHOS complexes were obtained from Thermo Fisher Scientific (1:1000, United States). Antibody *p*-Ser was obtained from Abcam (1:1000, AP0932, China). Goat anti-rabbit/mouse secondary antibody was purchased from ZSGB-BIO (Beijing, China). Nebivolol was purchased from Selleck (Shanghai, China). 4-Phenylbutyrate (4-PBA) was purchased from Sigma-Aldrich (St. Louis, MO, United States). MitoTracker Red was obtained from Solarbio (Beijing, China).

### Cell Counting Kit-8 Assay

Briefly, cells were seeded in 96-well plates at a density of 2 × 104 cells per well and treated with different concentrations of nebivolol (0, 1, 5, 10, 15, 20, and 50 μM) for 24 h. The cell proliferation rates were determined by CCK-8 solution and incubated at 37°C for 1 h. Absorbance was measured at a wavelength of 450 nm using a plate reader.

### Colony Formation Assay

HSC-3 and HN12 cells were cultured in 6-well plates at a density of 500 cells per well. After being treated with different concentrations of nebivolol (0, 5, 10, and 15 μM) for 24 h, cells were incubated in a humidified atmosphere of 5% CO_2_ at 37°C for 14 days until colony formation was observed. Then, cells were fixed in 4% paraformaldehyde for 30 min and stained with 1% crystal violet for 30 min. The stained cells were captured by stereoscopic microscope.

### Flow Cytometric Assays

Flow cytometry was used to measure cell apoptosis and the cell cycle distribution. Cell apoptosis assay was evaluated by Annexin V-FITC apoptosis detection kit (Nanjing KeyGen Biotech Co. Ltd., Nanjing, China) according to the manufacturer’s instructions. The cell cycle distributions were evaluated by a cell cycle detection kit (Nanjing KeyGen Biotech Co. Ltd., Nanjing, China) according to the manufacturer’s instructions.

### TUNEL Assay

The apoptotic cells were evaluated by using DeadEnd^TM^ Fluorometric TUNEL system (Promega) according to the manufacturer’s instructions. The percentage of apoptotic and nonapoptotic cells was observed by fluorescent microscope.

### Transmission Electron Microscopy Analysis

HSC-3 and HN12 were cultured in a 10 cm dish and incubated with vehicle or 10 μM nebivolol for 24 h. The cells were harvested and fixed in a 3% glutaraldehyde solution. Then, cells were prefixed in Karnovsky’s solution and washed with cacodylate buffer. After dehydration with alcohol, the cells were embedded in Poly/Bed 812 resins and polymerized and observed under an electron microscope (EM 902A, Zeiss, Oberkochen, Germany).

### Mitochondrial Morphology Analysis

OSCC cells were cultured in a 20 mm glass plate and treated with or without nebivolol for 24 h. Later, the cells were stained with 1 nM MitoTracker Red and 10 nM Hoechst at 37°C for 15 min. The images were captured by Confocal Olympus fluorescence microscope.

### Quantitative Real-Time PCR

Total RNA was gathered by TRIzol reagent (Invitrogen) according to the manufacturer’s instructions. The concentration of total RNA was measured by NanoDrop One (Thermo Fisher Scientific). cDNA was prepared from 2 μg RNA, which utilized PrimeScript RT Reagent kit with gDNA Eraser (Takara). qRT‐PCR was performed using SYBR Mix on a Bio‐Rad CFX96 Real‐Time System. The relative mRNA expressions were normalized by the GAPDH gene and calculated by using the 2^−∆∆Ct^ method when compared to the control groups. The relative primers’ sequences were listed in Supplementary Table 1.

### Measurement of Mitochondrial Mass

The mitochondrial mass was evaluated by using acridine orange 10-nonyl bromide (NAO, HY-D0993). Cells were treated with or without 10 µM nebivolol for 24 h. Later, the treated cells were gathered and incubated with 5 µM NAO for 20 min and analyzed by using the flow cytometer (Beckman FC500, Brea, CA, United States).

### Mitochondrial Respiration

The OSCC cells were reseeded in 24-well microplates which were treated with and without 10 µM nebivolol for 2 h. The corresponding treated cells’ oxygen consumption rate (OCR) was measured by Seahorse XFp Extracellular Flux Analyzer (Seahorse Bioscience). The sequential final concentrations of injected substances were 2.5 μM oligomycin, 1 μM FCCP, 1 μM rotenone, and antimycin. Each group was determined five times and normalized according to the number of cells.

### Intracellular Reactive Oxygen Species Assay

The cells were incubated with or without 10 μM nebivolol for 24 h and then harvested and resuspended in PBS. The cells were incubated with 5 μM H2DCFDA for 30 min in the dark. Finally, the fluorescence intensity of the 2,7-dichlorofluorescein dye was quantified by using a plate reader.

### Mitochondrial Membrane Potential Assay

Cells were treated with or without 10 µM nebivolol for 24 h. Then, cells were gathered to measure mitochondrial membrane potential by using Mitochondrial Membrane Potential Assay Kit (C2006, Beyotime, Shanghai, China) according to the manufacturer’s instructions. The treated cells were gathered and analyzed by using the flow cytometer (Beckman FC500, Brea, CA, United States).

### Western Blot Analysis

The cells were stimulated with different concentrations of nebivolol. The proteins were gathered in each group. The instructions of western blot analysis were described elsewhere. The primary antibodies were described above. The results were visualized by using an enhanced chemiluminescence Western-blotting detection reagent (Millipore, Billerica, MA, United States).

### siRNA Transfection

The small interfering RNAs (siRNAs) were designed and obtained by Ribobio (Guangzhou, China) and were transfected with Lipofectamine RNAiMAX reagent (Invitrogen) according to the manufacturer’s protocol. The sequences of siRNA were as follows: si iNOS1: GTG​CTG​TAT​TTC​CTT​ACG​A and: si iNOS2: GAA​GCG​TAT​CAC​GAA​GAT.

After transfection for almost 48 h, the groups were treated with or without nebivolol for 3 h or 12 h, and the corresponding gene and protein expressions were evaluated by quantitative real-time PCR (qPCR) and Western blot.

### Lentiviral Transfection

For gene knockdown, lentiviruses knockdown ATF4 or negative vectors were designed and purchased from JiKai (Shanghai, China). The cells were transfected with lentiviruses for 72 h and selected with 2 μg/ml puromycin (Sigma). The stable expression cell lines were treated with nebivolol for 12 h or 24 h. The corresponding gene expressions were evaluated by qPCR, and the cell viability was detected by CCK-8 assay.

### Patient Tissue Samples

The OSCC patients were diagnosed by two experienced pathologists. OSCC tissue samples were obtained from the Hospital of Stomatology, Sichuan University, with informed consent from patients. The tissues were collected and used in accordance with a protocol approved by the Human Research Ethics Committee of the West China Hospital of Stomatology, Sichuan University.

### 4NQO‐Induced Mice OSCC Model

Twenty female C57BL/6 mice, weighing 16–20 g, 4–6 weeks old, were used to induce oral carcinomas. Protocols for animal experiments were approved by the Animal Care and Use Committee, State Key Laboratory of Oral Diseases, Sichuan University, in compliance with the guidelines approved by the National Institute of Health (NIH) in the United States. The mice were housed in the specific pathogen-free (SPF) unit of the animal facility in accordance with institutional guidelines. 4NQO was used to establish the oral carcinomas model. The mice were fed up with 100 μg/ml 4NQO chemistry medicine to induce tongue tumor model. Meanwhile, animals were randomly allocated into different groups and were treated with a single daily intraperitoneal injection of 25 mg/kg nebivolol or 125 mg/kg 6OHDA every two weeks. After 24 weeks, the mice were euthanized, and the mice tongues were gathered to prepare for immunohistochemistry analysis.

### Tumor Xenograft Model

HSC-3 cells were gathered, washed, and resuspended in DMEM, which were subcutaneously injected (2 x 10^5^ cells (200 μl)) into mucosal tissue of the tongue area of athymic BALB/c nude mice. After 2 weeks of implantation, mice were anesthetized and the tumor was measured. Meanwhile, animals were randomly allocated into different groups and were treated with an intraperitoneal injection of 20 mg/kg nebivolol or the same dose of 0.9% NaCl buffer every three days. Then, the engrafted tumors were fixed in 4% paraformaldehyde overnight, which were prepared for the following histological analyses and morphologic analyses.

### Immunohistochemistry

The distribution of β-Ⅲ-tubulin was evaluated by IHC in the 4NQO‐induced mice OSCC model and tumor xenograft model. IHC was used to determine tumor-related markers in tumor xenograft tissues and the 4NQO‐induced mice OSCC model. The standard protocol for IHC was similar to the previous one. The images were scanned by Aperio Digital Pathology Systems (ScanScope GL; Aperio Technologies).

### Immunofluorescence

HSC-3 and HN12 cells were treated with or without nebivolol for 12 h. Briefly, the cells were fixed with 4% paraformaldehyde. Then, 0.1% Triton-100 punched the cells for 2 min, which were blocked by 10% FBS for 30 min. Subsequently, cells were incubated overnight with anti-ATF4 (1:100) at 4°C. The next day, Alexa Fluor 488-conjugated goat anti-rabbit (1:750, Invitrogen) was used as a fluorescent second antibody. The cell nuclei were stained with 4,6-diamino-2-phenylindole (DAPI). The images were captured by confocal laser-scanning microscopy (CLSM, Tokyo, Japan and Olympus, FV3000, Japan).

To identify the nebivolol effect on tumor angiogenesis, CD31 antibody was used to stain specimens in mice oral tumor model. Alexa Fluor 488-conjugated goat anti-rabbit (1:750, Invitrogen) was used as a fluorescent second antibody. The cell nuclei were stained with DAPI. The images were captured by an Olympus fluorescence microscope.

### Mitochondrial Enzyme Activities

HSC-3 and HN12 cells were treated with or without nebivolol for 12 h. Then, cells were gathered to measure the activities of complexes I by using Mitochondrial Respiratory Chain Complex Ⅰ Activity Assay Kit (D799471, Sangong, Shanghai, China) according to the manufacturer’s instructions. Absorbance was measured at A_340_ by using spectrophotometric (Agilent Cary 100). The concentrations of protein were relatively quantified by BCA kit (Thermo Fisher Scientific).

### Statistical Analysis

All experiments were carried out independently at least three times. The results are calculated as the means ± standard deviation (SD) using GraphPad Prism software (version 7.2; GraphPad Software, Inc., La Jolla, CA, United States). The differences between the experimental groups and controls were assessed by single-factor analysis of variance (ANOVA) and Student’s *t*-test. A *p*-value less than 0.05 (*p* < 0.05) was regarded as a statistically significant result.

## Results

### Nerves Infiltrated in the Microenvironment of OSCC

**FIGURE 1:**
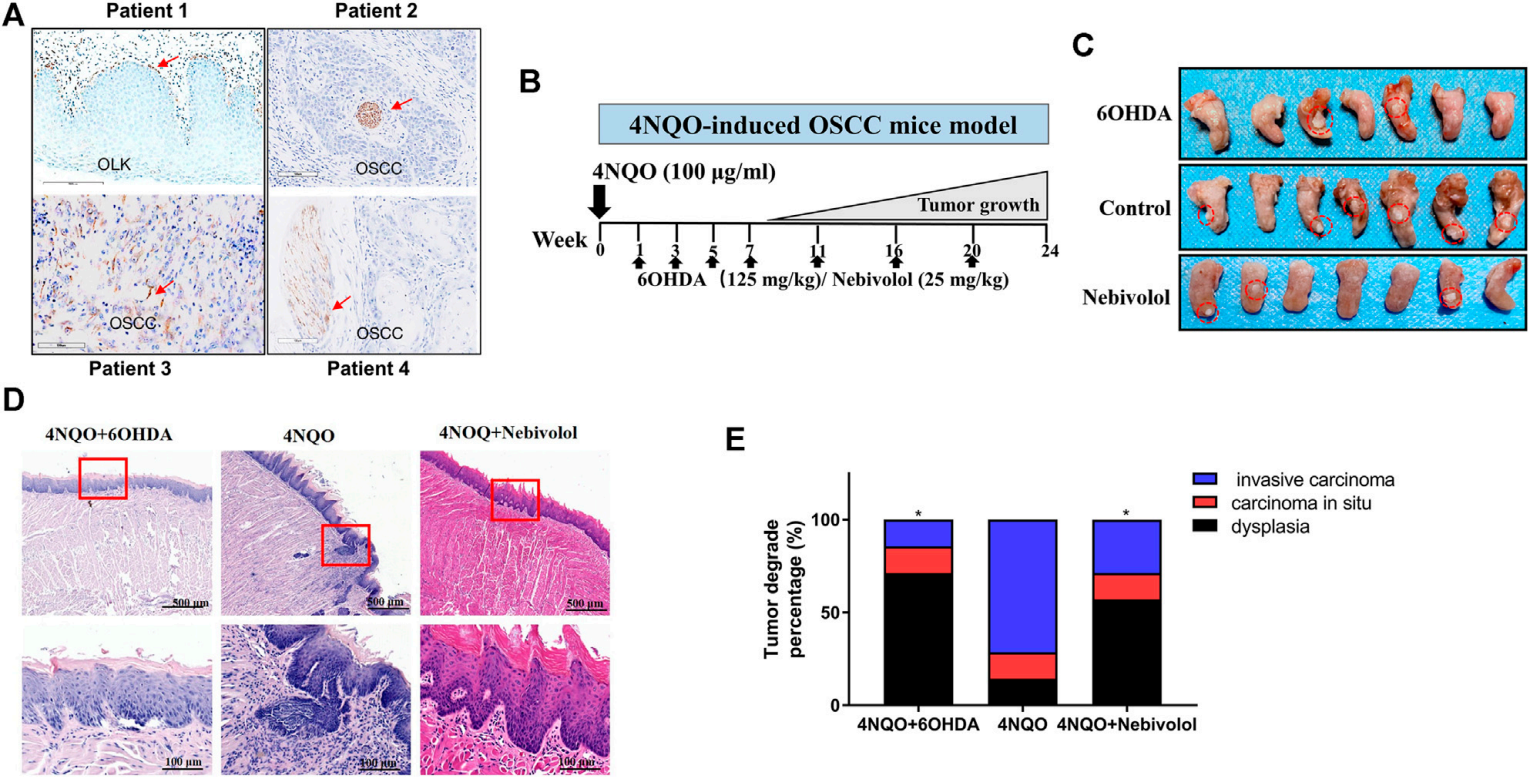
The distribution of nerves in OSCC tissues and 6OHDA halted the growth of tumors in the 4NQO-induced tumor mice model. **(A)** The distribution of β-Ⅲ-tubulin protein in OLK and OSCC tissues. The red arrows showed the positive staining. **(B)** The pattern of 4NQO-induced tumor mice model. **(C)** Representative images of isolated 4NQO-induced tumors treated with vehicle or nebivolol or 6OHDA for 24 weeks. **(D)** Representative images of 4NQO-induced tumor in three groups in HE staining. The red boxes’ images were magnified at the bottom of the images. **(E)** The histopathological degrades of tumor lesions were analyzed. Data represent the mean ± SD of three replicate independent experiments. The asterisk (*) indicates a significant difference compared to the control group (*p < 0.05).

In order to determine the distribution of nerve fibers in OSCC tissues, the neuromarker β-Ⅲ-tubulin was used as the target protein. IHC experiments were performed on the pathological tissue sections of the patients who had been clinically diagnosed with OSCC or OLK. Representative images of β-Ⅲ-tubulin staining of OLK and OSCC samples are shown in Figure 1A. Nerves existed in OSCC tissue. In addition, the tumor tissue wrapped the nerve bundles like a sleeve, suggesting that the nerve is a component of the TME.

### 6OHDA Halted 4NQO-Induced Oral Mucosa Carcinogenesis *In Vivo*

The 4NQO-induced OSCC model was established. 6OHDA was injected periodically in the intraperitoneal cavity (Figure 1B). After 24 weeks, the tissues of the tongue lesions and important organs were gathered for histological examination. As shown in Figure 1C, when compared to the control groups, the tumor sizes in the 6OHDA groups were significantly reduced. Besides, the representative images of tongue tumors were exhibited in HE staining (Figure 1D). In addition, the lesions were analyzed in degrades. The percentage of dysplasia in the 6OHDA administration groups was significantly increased when compared to the 4NQO-induced groups. However, the percentage of tumor lesions was apparently reversed when compared to the percentage of dysplasia (Figure 1E). Collectively, these results demonstrated that using 6OHDA to destroy the sympathetic nervous system could delay the progression of OSCC. Although 6OHDA halted cancer growth in tongue mice, the organs suffered from toxicity in different degrees when compared to the control groups.

In addition, IF staining was performed to analyze the expression of CD31 after being treated with nebivolol in the mice oral tumor model, which aims to analyze the nebivolol effect on tumor angiogenesis. As shown in Supplementary Figure 1, the expression of CD31 (green) was apparently reduced in nebivolol-treated mice when compared to the control group. This indicated that nebivolol triggered significant inhibition of tumor angiogenesis (Supplementary Figure 1A).

### Nebivolol Halted Cancer Growth in Orthotopic Tongue Tumor Mice and 4NQO-Induced Tongue Mice

**FIGURE 2:**
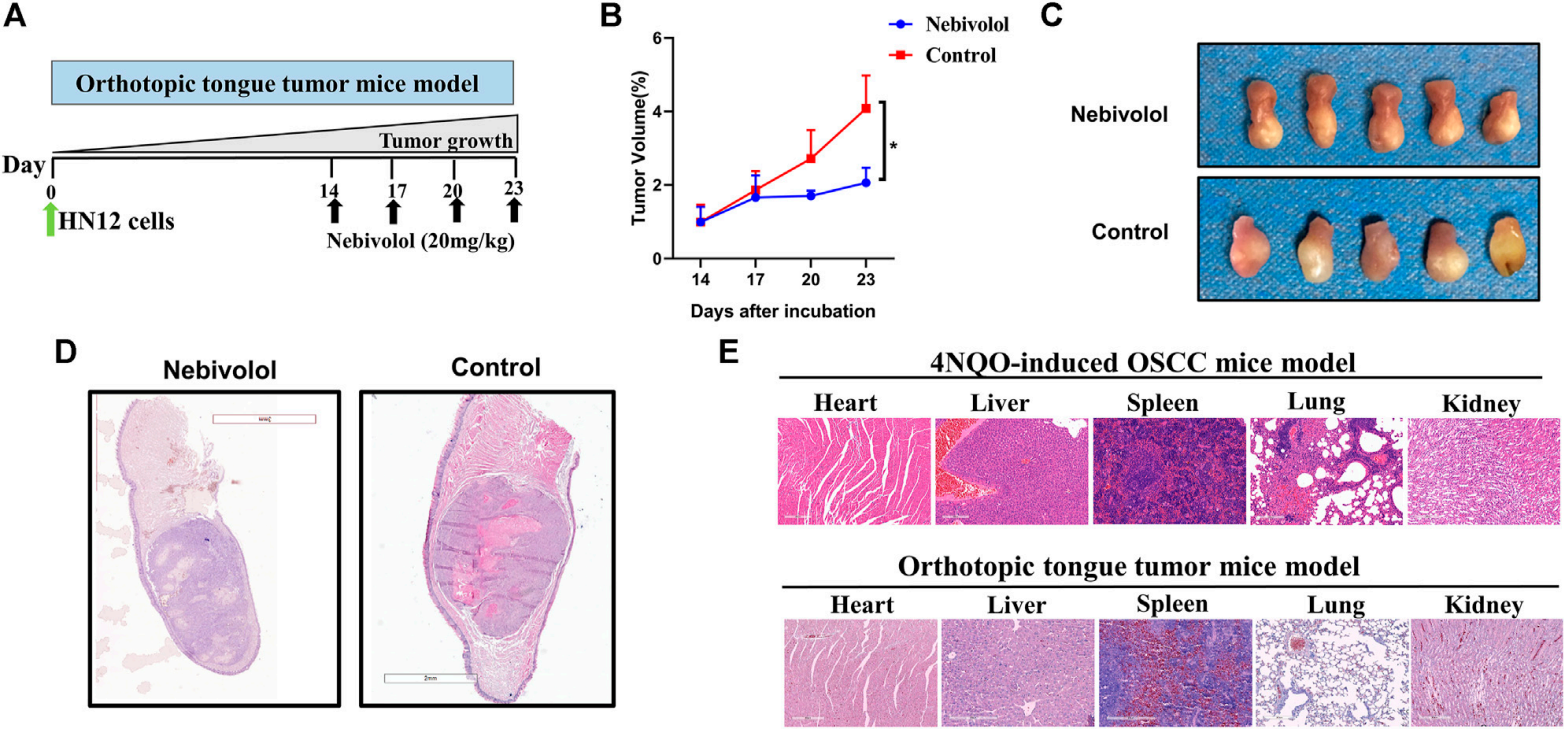
Nebivolol halted cancer growth in orthotopic tongue mice and chemically induced tongue mice. **(A)** The pattern of orthotopic tongue tumor mice model. **(B)** Tumor volume was determined at the indicated time point and the growth curves from two groups of mice during 3 weeks were made in the xenograft tumor model. **(C)** Representative images of isolated xenograft tumors treated with vehicle or nebivolol for 3 weeks. **(D)** Representative HE staining image of isolated xenograft tumors after being treated with vehicle or nebivolol for 3 weeks. **(E)** HE stained images of liver, heart, spleen, lung, and kidney in tumor tissues of the control groups and the nebivolol-treated groups from two mice models. Data represent the mean ± SD of three replicate independent experiments. The asterisk (*) indicates a significant difference compared to the control group (*p < 0.05).

In order to discover a drug that could be alternated to 6OHDA, nebivolol was used, which could not only halt cancer growth but also show inapparent toxicity to organs. As shown in Figure 2A, the orthotopic tongue tumor mice were established and nebivolol was administrated at appointed times. The growing trend of tumor sizes was observed between the two groups in the tumor xenograft model. The growth of tumors in the nebivolol treatment group was significantly slower than that in the control group (Figure 2B). The representative images of tongue tumors were exhibited (Figure 2C). Moreover, the same trend of tumor growth was observed in the 4NQO‐induced mice OSCC model (Figure 1C). Besides, the representative images of tongue tumors were exhibited in HE staining (Figure 2D). In addition, the lesions were analyzed and histopathological grades evaluation were performed. The percentage of dysplasia in the 6OHDA administration groups was significantly increased when compared to the 4NQO-induced groups. However, the percentage of tumor lesions was apparently reversed when compared to the percentage of dysplasia (Figure 1E). These resulted illustrated that nebivolol halted cancer growth in the orthotopic tongue tumor mice model and the 4NQO-induced tongue mice model without toxicity in the organs (Figure 2E).

### Nebivolol Inhibited the Growth in OSCC Cells

We used RT-qPCR to detect the expression levels of each adrenergic receptor subtype gene (ADRβ1, ADRβ2, and ADRβ3) in OSCC cells. The cDNAs of normal human keratinocyte (HOK) and OSCC cell lines (Cal27, HSC-3, HSC-4, HN4, and HN12) were collected, and the expression levels and differences of the corresponding receptor genes in each cell line were detected by RT-qPCR. Experimental results showed that the expression levels of neurotransmitter receptor genes in various OSCC cell lines are significantly different from those of normal keratinocytes. Compared with normal human keratinocytes HOK, the β1 receptor mRNA of HN12, HSC-3, and HSC-4 in OSCC cells is highly expressed. However, there was no significant difference among the expression of ADRβ2 and ADRβ3 receptors in OSCC cells and HOK cells (Supplementary Figure 1B, C, D).

**FIGURE 3:**
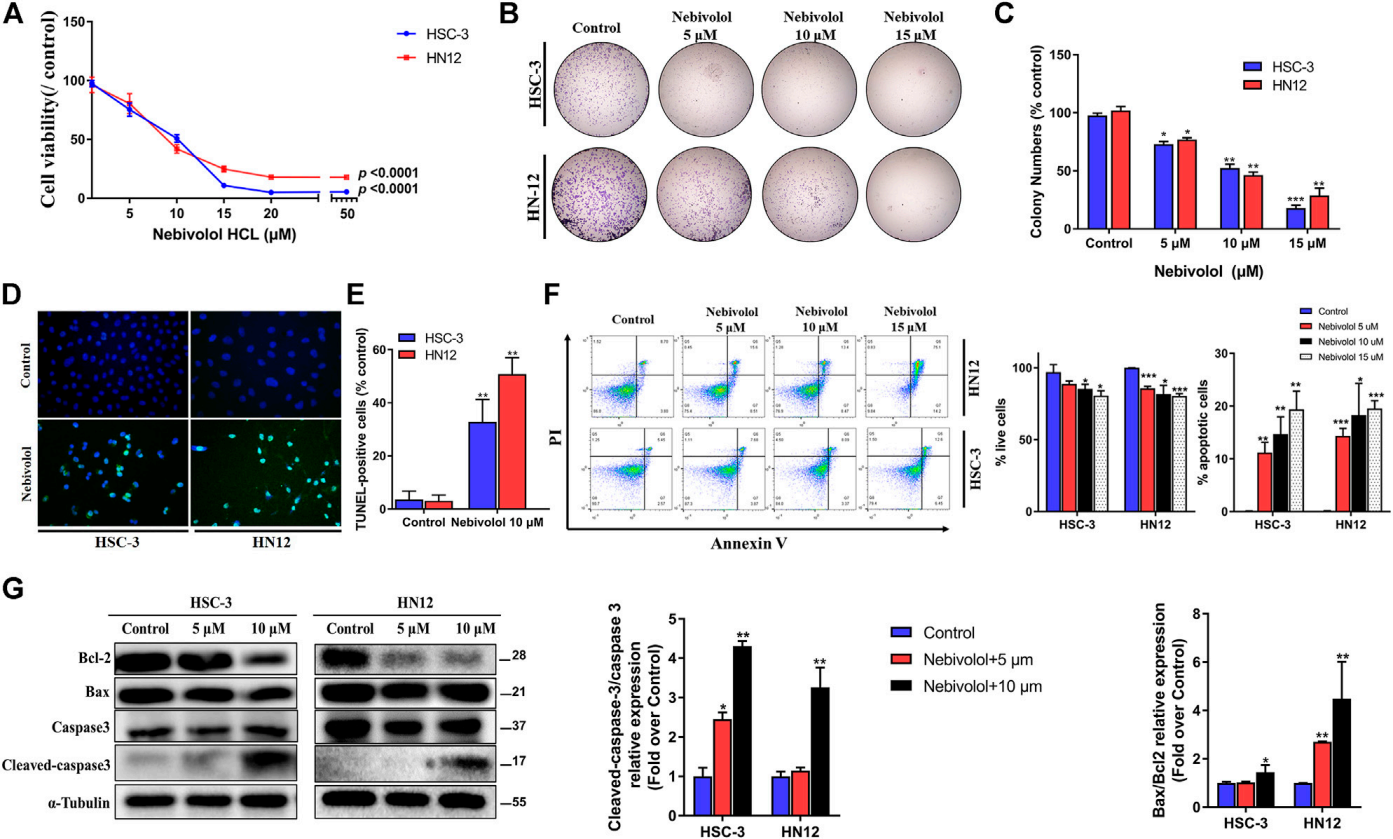
Nebivolol inhibited the growth in OSCC cells. **(A)** The CCK-8 assay was used to determine the cytotoxicity of nebivolol on HSC-3 and HN12 cells at different concentrations for 24 h. **(B, C)** Colony formation assay of HSC-3 and HN12 cells treated with the indicated concentrations of nebivolol. Representative images **(B)** and quantification of colonies **(C)** were shown. **(D, E)** TUNEL assay in nebivolol-treated cells. Representative images **(D)** and quantification of TUNEL-positive cells **(E)** were shown. Scale bar, 100 μm. **(F)** Cell death assay using Annexin V/PI staining and flow cytometric to determine the effects of nebivolol on HSC-3 and HN12 cells death. The proportion of live cells and apoptotic cells was quantified. **(G)** Western blotting of total and cleaved-caspase 3, Bcl-2, and Bax in OSCC cells after being treated with the indicated concentrations of nebivolol for 24 h. The Bax/Bcl2 and cleaved/total caspase 3 from the blots were quantified. Data represent the mean ± SD of three replicate independent experiments. The asterisk (*) indicates a significant difference compared to the control group (*p < 0.05, **p < 0.01, and ***p < 0.001).

To examine the effect of nebivolol on the growth of OSCC cells, nebivolol was applied on HSC-3 and HN12 cells. As shown in Figure 3A, the cell viability significantly decreased with the increasing concentration of nebivolol in OSCC cells at 24 h. Nebivolol induced a dose-dependent cytotoxic effect in the OSCC cells. Similarly, the number of HSC-3 and HN12 cell colonies was significantly reduced after being treated with an increasing concentration of nebivolol. The clonogenicity of OSCC cells was significantly inhibited in a dose-dependent manner (Figures 3B,C).

In order to illustrate the association of apoptosis and antitumor activities induced by nebivolol, the apoptotic ratio was evaluated. As shown in Figures 3D,E, there were a significantly elevated percentage of apoptotic cells when treated with an increased concentration of nebivolol for 24 h. Consistently, after being treated with nebivolol for 24 h, flow cytometric double staining by Annexin V-FITC apoptosis detection showed an obvious effect on apoptosis induction in OSCC cells. With the increasing concentrations of nebivolol treatment, the proportion of live cells was significantly decreased. Adversely, the proportion of apoptotic cells was significantly increased in OSCC cells when compared to the control groups (Figure 3F). Furthermore, after nebivolol treatment, the ratio of cleaved-caspase 3 and procaspase 3 as well as Bcl-2 and Bax was significantly increasing (Figure 3G). To sum up, these data illustrated that nebivolol inhibited the growth of OSCC cells by inducing apoptosis.

### Nebivolol Activated Apoptosis *via* Endoplasmic Reticulum (ER) Stress Signaling Pathway in OSCC Cells

**FIGURE 4:**
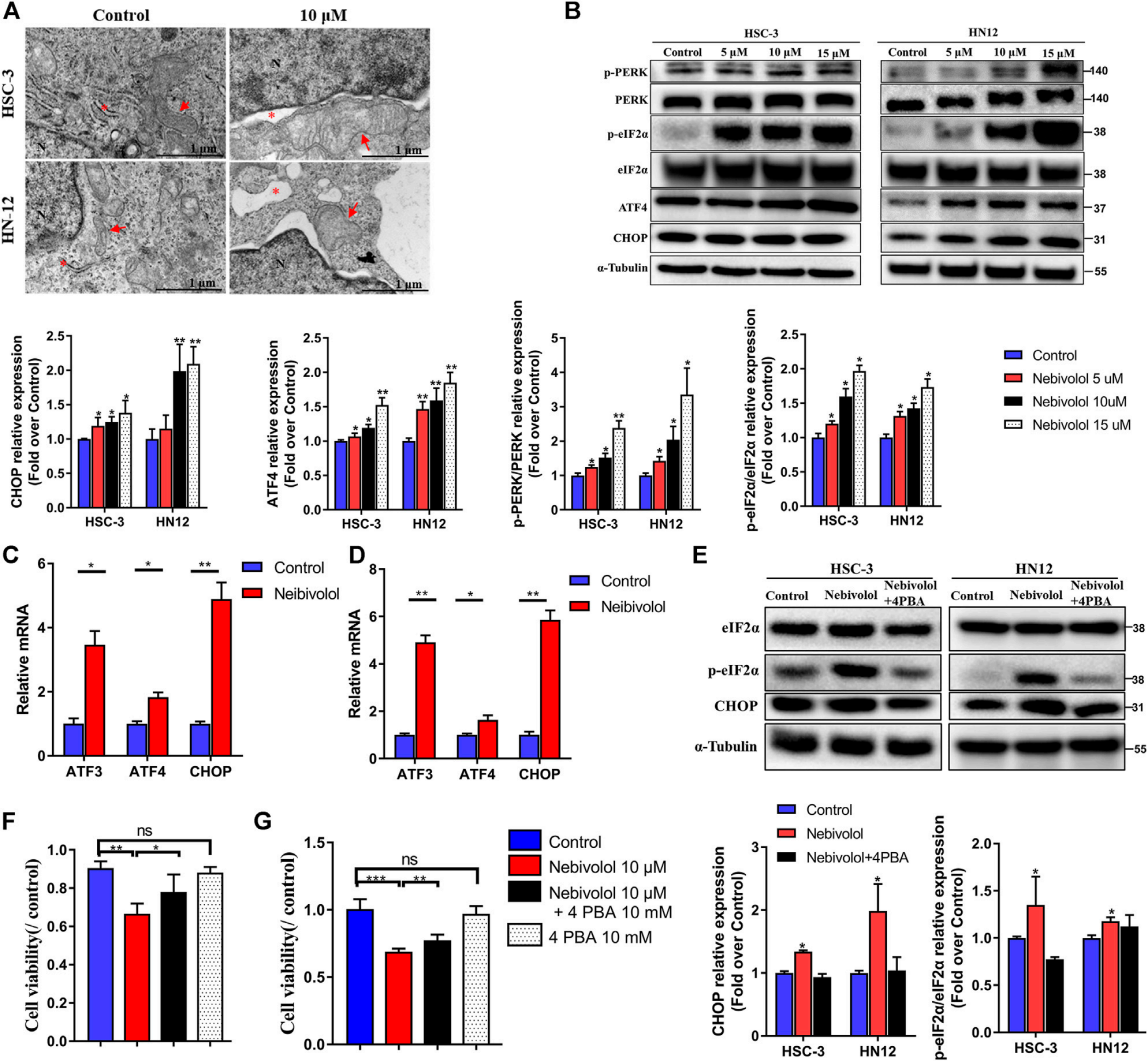
Nebivolol activated apoptosis *via* ER stress signaling pathway in OSCC cells. **(A)** Representative images of cytoplasmic vacuoles detected by TEM in OSCC cells after being treated with nebivolol and controls. Massive cytoplasmic vacuolization was visible which was derived from enlarged ER and mitochondria after being treated with isorhamnetin. Red arrows: ER; Red asterisks: mitochondria. Scale bar = 1 μM. **(B)** Western blotting of ER stress markers was examined in OSCC cells after being treated with the indicated concentrations of nebivolol. The p-PERK, *p*-eIF2α, ATF4, and CHOP from the blots were quantified. **(C, D)** The qRT-PCR analysis confirmed that ER stress gene expression was upregulated in HSC-3 cells **(C)** and HN12 cells **(D)**. **(E)** Western blotting of total and phosphorylated eIF2α and CHOP in OSCC cells after treatment with or without nebivolol in the presence or absence of 4-PBA. The *p*-eIF2α and CHOP from the blots were quantified. **(F–G)** The CCK-8 assay was used to determine the cell viability of nebivolol in the presence or absence of 4-PBA on HSC-3 **(F)** and HN12 cells **(G)**. Data represent the mean ± SD of three replicate independent experiments. The asterisk (*) indicates a significant difference compared to the control group (*p < 0.05, **p < 0.01, and ***p < 0.001).

After being treated with nebivolol in OSCC cells, there were abundant cytoplasmic vacuoles observed apparently when compared to the control groups (Figure 4A). TEM was employed to detect the origination and cell ultrastructure of the observed vacuole. Compared to the control groups, there were numerous vacuoles detected after being treated with nebivolol. Furthermore, the vacuoles were derived from swollen portions of the ER and megamitochondria (Figure 4A). These results demonstrated that nebivolol-induced cytoplasmic vacuoles were derived from swollen ER and mitochondria, which might be associated with nebivolol-induced apoptosis in OSCC cells.

Much evidence has approved that ER stress was closely related to apoptosis (Hetz and Papa, 2018). In order to reveal the activated signal pathway of nebivolol-induced apoptosis in OSCC cells. The proteins expressions of ER stress markers were detected. As shown in Figure 4B, the results revealed that the proportion of phosphorylated protein level, p-PERK, and p-eIF2α significantly gradually elevated after increased concentrations treatment of nebivolol when compared to the corresponding protein level in the control groups. While the protein expression of PERK and eIF2α was still maintained at the same level, the protein level ratio of phosphorylated proteins compared to corresponding total proteins significantly increased after treatment with an increased dose of nebivolol. Subsequently, the significantly increased expression of ATF4 and CHOP was also observed after being treated with an increased dose of nebivolol when compared to the control groups (Figure 4B). In addition, the gene expression of the ER stress downstream pathway was also supported by the activated signal pathway (Figures 4C,D). These data revealed that the PERK-eIF2α-ATF4-CHOP axis signal pathway was activated when treated with nebivolol in OSCC cells. To ascertain that nebivolol-induced ER stress led to apoptosis, cells were treated with the ER stress inhibitor 4-PBA and nebivolol. 4-PBA significantly decreased nebivolol-induced overexpression of the ER stress markers (Figure 4E). Meanwhile, the cell viability was significantly enhanced after the combined treatment with nebivolol and 4-PBA when compared to the nebivolol groups (Figures 4F,G). These results demonstrated that nebivolol activated apoptosis *via* ER stress signaling pathway in OSCC cells.

### Nitric Oxide Synthase Accumulation Contributed to Nebivolol-Induced ER Stress in OSCC Cells

**FIGURE 5:**
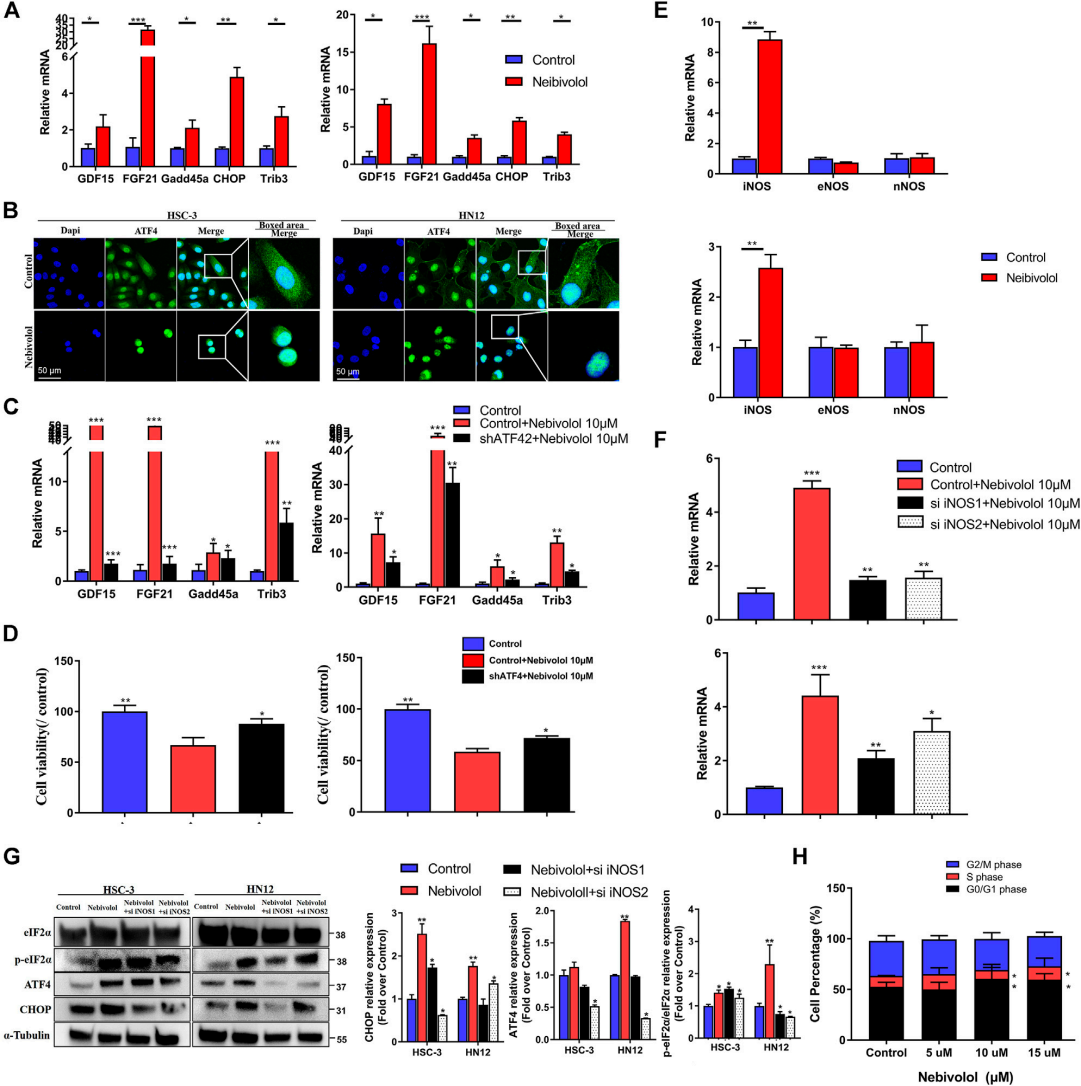
Nitric oxide synthase (iNOS) accumulation contributed to nebivolol-induced ER stress in OSCC cells. **(A)** The qRT-PCR analysis confirmed that the ISR gene was upregulated after being treated with nebivolol in OSCC cells. **(B)** Immunofluorescent images showing the translocation of ATF4 in OSCC cells after induction with nebivolol. The OSCC cells were stained with markers of the nucleus (DAPI, blue). The images are based on three independent experiments observed by CLSM. **(C)** The qRT-PCR analysis confirmed that the ISR gene was downregulated in the shATF4-transfected cells after being treated with nebivolol for 12 h when compared to the nebivolol-stimulated OSCC cells. **(D)** The cell viability were increased in the shATF4-transfected cells groups after being treated with nebivolol for 12 h when compared to the alone nebivolol-treated groups. **(E)** qRT-PCR analysis showed that iNOS gene expression was enhanced after being treated with nebivolol in OSCC cells. (F) The qRT-PCR analysis confirmed that iNOS gene expression was decreased in the nebivolol-induced si iNOS groups when compared to the nebivolol-stimulated OSCC cells. **(G)** Western blotting of total and phosphorylated eIF2α, ATF4, and CHOP in OSCC cells in the nebivolol-induced si iNOS groups and the nebivolol-induced groups. The *p*-eIF2α, ATF4, and CHOP from the blots were quantified. **(H)** Cells were treated with the indicated concentration of nebivolol for 12 h and the cell cycle distributions were detected by flow cytometry assay. Data represent the mean ± SD of three replicate independent experiments. The asterisk (*) indicates a significant difference compared to the control group (*p < 0.05, **p < 0.01, and ***p < 0.001).

The integrated stress response (ISR) is a countermeasure that is initiated when the cell is damaged from outside or inside. To investigate whether nebivolol induces ISR gene expression, the qPCR was applied. As shown in Figure 5A, the ISR genes were significantly increased in the presence of nebivolol for 12 h. As the typical transcription factor of ISR response, ATF4 could start a series of gene expressions that respond to stress (Pakos-Zebrucka et al., 2016). After being treated with nebivolol, the expressions of ATF4 significantly increased and transferred from cytoplasm to cell nucleus when compared to the control groups (Figure 5B).

To identify the explicit effect of ATF4 activation on ISR gene expression in nebivolol-treated OSCC cells, the knockdown ATF4 lentivirus was used in OSCC cells. The ISR genes were detected. As shown in Figure 5C, as compared to the nebivolol groups, ATF4 knockdown cells suppressed ISR genes expression when treated with nebivolol for 12 h. Meanwhile, the cell viability was significantly enhanced in ATF4 knockdown cell lines treated with nebivolol for 12 h when compared to the nebivolol-induced groups (Figure 5D). These data demonstrated that translation of ER and mitochondrial regulated nebivolol-mediated ISR gene expression through ATF4 activation.

To further study the upstream signaling pathway of nebivolol-induced ER stress, the NO-associated synthases were analyzed. The marked increased iNOS expressions were observed after being treated with nebivolol for 3 h, which indicated that nebivolol triggered the expression of iNOS to induce ER stress in OSCC cells (Figure 5E). In order to elucidate the role of iNOS in nebivolol-induced ER stress, the cells were then treated with nebivolol in the presence or absence transfected with si iNOS. The gene expression of iNOS was significantly inhibited in the nebivolol-induced si iNOS groups (Figure 5F). Furthermore, the downstream ER stress markers were also detected. The protein expressions of ATF4 and CHOP significantly decreased in the nebivolol-induced si iNOS groups when compared to the nebivolol-treated groups. The proportion of p-eIF2α protein expression significantly decreased in the nebivolol-induced si iNOS2 groups when compared to the nebivolol-treated groups (Figure 5G). Overall, these data revealed that nebivolol triggered the expression of iNOS to activate ER stress signal pathway in OSCC cells.

In addition, the results showed that the proportion of HN12 cells was significantly increased in the S phase and reduced in the S and G2/M phase following treatment with 10 and 15 μM nebivolol when compared to the proportions in the corresponding phases of the control group cells. Thus, nebivolol significantly arrested cell cycle progression at G0/G1 phase (Figure 5H).

### Nebivolol Increased Reactive Oxygen Species Level to Induce Apoptosis *via* Stimulating Mitochondrial Dysfunction in OSCC Cells

**FIGURE 6:**
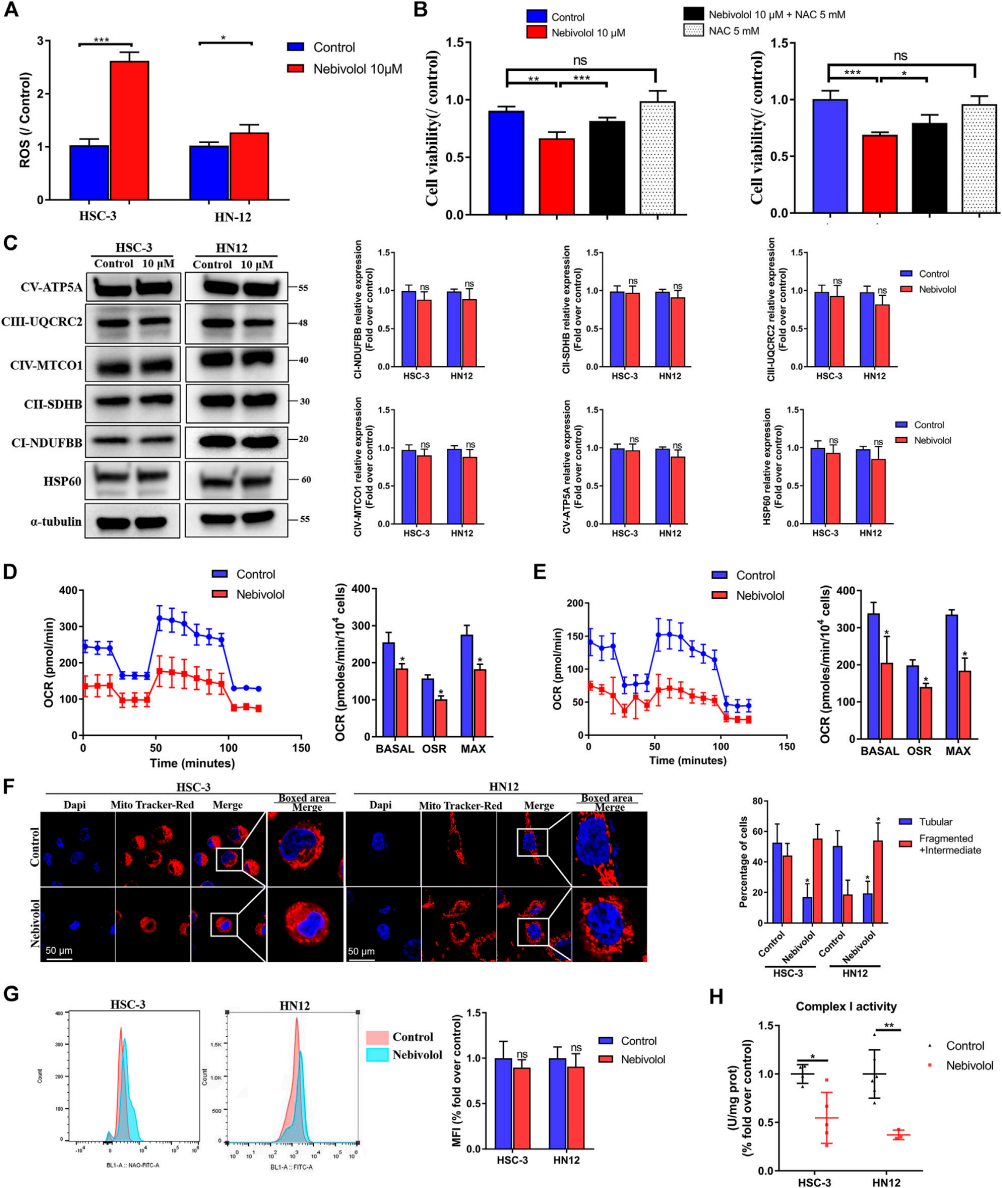
Nebivolol increased ROS level to induce apoptosis *via* a disturbing mitochondrial function in OSCC cells. **(A)** The ROS level was enhanced after nebivolol induction for 24 h in OSCC cells. **(B)** The CCK-8 assay was used to determine the cell viability of nebivolol in the presence or absence of pretreatment of NAC on OSCC cells for 24 h. **(C)** Effects of OXPHOS proteins expression in OSCC cells after being treated with 10 μM nebivolol for 12 h. The data are representative of five independent experiments. The CI-NDUFBB, CII-SDHB, CIII-UQCRC2, CIV-MTCO1, CV-ATP5A, and HSP60 from the blots were quantified. **(D)** Oxygen consumption rate (OCR) was detected when treated with or without 10 μM nebivolol in the HSC-3 cells. The basal respiration, ATP production, and maximal respiratory capacity were quantified. **(E)** OCR was detected when treated with or without 10 μM nebivolol in the HN12 cells. The basal respiration, ATP production, and maximal respiratory capacity were quantified. **(F)** HSC-3 and HN12 cells were treated with or without 10 μM nebivolol for 24 h and then incubated with MitoTracker Red. Images were obtained by fluorescence microscopy. Scale bar, 50 μm. The mitochondrial morphology was assessed from five cells on three different slides. **(G)** Cells were treated with the 10 μM nebivolol for 24 h and the mitochondrial mass was detected by NAO fluorescence which was quantified by mean fluorescence intensity (MFI). **(H)** Cells were treated with the 10 μM nebivolol for 12 h and enzymatic activity of complex I was detected. Data represent the mean ± SD of three replicate independent experiments. The asterisk (*) indicates a significant difference compared to the control group (*p < 0.05, **p < 0.01, and ***p < 0.001).

Although the inhibitor 4-PBA significantly rescued the cell viability when treated with nebivolol for 12 h, the inhibitor 4-PBA could not recover cell viability after being treated with nebivolol for 24 h. In order to reveal the underlying mechanism of nebivolol-induced apoptosis after ER stress signal pathway being activated in OSCC cells, we examined the possible involved ROS levels. As shown in Figure 6A, the ROS levels were apparently enhanced after being treated with nebivolol for 24 h when compared to the control groups. Thus, it was confirmed that nebivolol could induce the production of ROS through mitochondrial activation in OSCC cells. However, there was no significant difference in mitochondrial membrane potential in HSC-3 and HN12 cells (Supplementary Figure 2A). An experiment was also implemented by using NAC to detect the effects of inhibitors on nebivolol-induced apoptosis. It could be demonstrated that, in Figure 6B, the cell viability was significantly rescued by a combination of NAC and nebivolol. Herein, the results revealed that the accumulated ROS induced apoptosis after being treated with nebivolol.

In order to demonstrate the reason for ROS accumulation, the protein expression of OXPHOS complexes was measured. There was no significant difference in proteins expression of OXPHOS complexes and mitochondrial structural protein expression HSP60 in the presence of nebivolol for 12 h when compared to the control groups (Figure 6C). These data indicated that protein expression of OXPHOS complexes’ subunits was not affected after being stimulated with nebivolol. While the OCR were sequentially measured, in the presence of nebivolol, the OCR was significantly reduced which indicated the dysfunction of mitochondrial functions. Specifically, the basal respiration, ATP production, and maximal respiration were significantly decreased in the nebivolol-treated groups when compared with the control groups (Figures 6D,E). To determine the reason for the dysfunction of mitochondria, we observed the morphology of mitochondria. After being stimulated with nebivolol, the morphology of mitochondria could be depicted as fragmented and intermediate bodies which changed from tubular mitochondrial filaments by using MitoTracker Red. Mitochondria in the nebivolol-treated groups were more fragmented and intermediate bodies and less tubular than those in the matched control groups (Figure 6F). These results verified that the changed mitochondrial morphology led to mitochondrial dysfunction which may affect mitochondrial respiratory function. Besides, NAO was used to detect mitochondrial mass. Flow cytometric data demonstrated that nebivolol exhibited no significant effect in mitochondrial mass when compared to the control groups (Figure 6G). The reduced mitochondrial respiration capacities were not related to OXPHOS complexes and the mitochondrial mass. Thus, the activity of OXPHOS complexes was detected to observe its relationship with mitochondrial respiration. Nebivolol significantly decreased the activity of complex I (Figure 6H). These indicated that the activity of OXPHOS complex I diminished after being stimulated with nebivolol which could affect the capability of ETC resulting in mitochondrial dysfunction. It is known that the phosphorylation of serine residues regulates the activity of proteins of OXPHOS (García-Bermúdez et al., 2015). The protein level of *p*-Ser was significantly downregulated in response to nebivolol treatment when compared to the control groups. However, we observed no significant difference in the protein expression of IF1 in OSCC cells which may indicate that nebivolol affects the activity of mitochondria and does not affect respiratory complex V in OSCC cells (Supplementary Figure 2B). Together, our data suggested that nebivolol disturbed mitochondrial capacity and increased ROS level to induce apoptosis in OSCC cells.

## Discussion

In this study, our preclinical data showed that using 6OHDA chemical denervation as well as using adrenergic blockade by nebivolol could halt the oral mucosa carcinogenesis. Our study suggested that repurposing nebivolol inhibits the growth of OSCC which could represent a credible value in clinical application. Remarkably, we explored the mechanism of the action of nebivolol against OSCC. Herein, the novel mechanism of nebivolol was activated by ER stress and mitochondrial dysfunction. Nebivolol promoted the accumulation of NO to activate the ER stress signal pathway which led to cell growth arrest and mitochondrial dysfunction. Further, the malfunctioning mitochondria with the impaired ETC could enhance the ROS production and finally induced OSCC cells' death. Our study first proved that the pharmacological blockade of adrenergic receptors was a suitable therapeutic strategy for OSCC treatment and repurposing nebivolol might represent an effective strategy to treat OSCC.

We discovered that nerves infiltrated the microenvironment of OSCC. After 6OHDA denervation, the progression of OSCC was decreased in the animal model. Indeed, a series of pioneering studies have shown that nerves played an essential role in the development of cancer. Nerves as regulators of cancer initiation and progression were gradually recognized (Zahalka and Frenette, 2020). The neurobiology of cancer in the TME is opening up a new perspective in oncology. Coarfa C confirmed that the interaction between cancer and nerve was critical to the progression of prostate cancer (Magnon et al., 2013). In oral cancer, nerves were a highly aggressive sign and a significant risk factor for local recurrence (Cracchiolo et al., 2018). In light of these advances, surgical or chemical denervation has become a novel therapeutic strategy for tumor treatment. However, nerves are essential for internal communication and proper physiological regulation of the body by connecting cells, organs, and the central nervous system. Denervation-based cancer therapies may face unpredictable risks. In this study, our results showed that denervation with 6OHDA significantly delays or inhibits the occurrence and development of OSCC. However, because of the potential cytotoxic effects of 6OHDA, its clinical application met with a high risk (Sandhu et al., 2009).

Thus, pharmacological blockade of neurotransmitter receptors may be a more suitable therapeutic strategy. In this regard, a third-generation β-blocker nebivolol was used to block the adrenergic receptors and its role as a potential anti-OSCC drug was evaluated. Our observation from the 4NQO-induced and orthotopic xenograft OSCC mice models as well as OSCC cell studies indicated that nebivolol could be repurposed as a potential candidate to suppress OSCC. Compared with neurotoxic drugs, like 6OHDA, this cardiocerebrovascular drug nebivolol has no major safety concerns, which makes it an attractive candidate for repurposing OSCC treatment. Actually, the article has reported that β-blockers were verified as an effective antitumor target in melanoma (Bustamante et al., 2019). Besides, in another study, Nuevo-Tapioles proved that nebivolol also could halt colon and breast tumor growth (Nuevo-Tapioles et al., 2020).

In this study, we confirmed that nebivolol could upregulate the expression of iNOS in OSCC cells and contribute to the activation of the NO signal pathway. Nebivolol was reported to beneficiate the response of the NO signal pathway to relieve hypertension (Varagic et al., 2010; Bertera et al., 2014). NO is synthesized by NO synthase (NOS) and as a multifunctional signaling molecule. NO is involved in the regulation of various pathophysiological processes such as blood vessels, nerves, and cytotoxicity (Fukumura et al., 2006). iNOS is widely present in tumor cells and has a dual role in tumors. Some authors argued that nebivolol could exert an antiapoptotic mechanism through downregulating the expression of iNOS in CsA-induced nephrotoxicity (El-Sheikh et al., 2019). Other studies also demonstrated that nebivolol protected renal injury by upregulating the expression of nNOS and reducing the effect of oxidative stress (Varagic et al., 2010). However, the typical mechanism of iNOS in tumor is still obscure (Somasundaram et al., 2019). A previous study reported the activation of iNOS could lead to S-nitrosylation of ER stress signal pathway and cause ER dysfunction in inflammation (Yang et al., 2015). We showed that ER stress was potently induced after nebivolol administration. Recently, activation of the ER stress signaling pathway for cancer treatment strategy has been proposed by several studies (Guo et al., 2010; Yang et al., 2015). In the condition of persisting chronic stimulus, the homeostasis of the ER would be broken, resulting in ER stress response. ER stress may contribute to cell growth arrest. In our study, we observed an increase in ER stress-related protein ATF4. We also confirmed that ATF4 initiated the procession of a series of downstream specific ISR-related mRNA translations. Targeting ISR could selectively break down the protein homeostasis in tumor cells and caused cells to undergo apoptosis, which also made ISR a potential chemotherapy target for various tumors (Magnon et al., 2013).

ER acts as a dynamic organelle that maintains cell homeostasis. When the homeostasis of the cells is levered, ER cannot regulate the disordered status; thereby, the downstream pathways are activated which would lead to cell death. With the accumulation of misfolded proteins in ER, the oxidative stress was activated and triggered the production of ROS (Lin et al., 2019). Our study observed that nebivolol significantly increased ROS levels and induced apoptosis in OSCC cells. Mitochondria are regarded as the typical sites of cellular respiration to induce the production of ROS. There is a vicious cycle among mitochondria, ROS, and carcinogenesis. The malfunctioning mitochondria enhance the production of ROS, which could promote cancer progression by impaired OXPHOS and/or nuclear DNA mutations (Yang et al., 2016). In this process, the examination of mitochondrial function was implemented.

We observed that the nebivolol showed no significant differences in the protein expression of OXPHOS complex subunits. Besides, the mitochondrial mass was not affected by nebivolol treatment. However, the fragmented and intermediate bodies changed mitochondria and the apparently reduced OCR demonstrated that disturbed mitochondria existed after stimulation by nebivolol. Subsequently, we showed that nebivolol significantly decreased the activity of complex I which could affect the capability of ETC, resulting in mitochondrial dysfunction. The major sites of OXPHOS, complexes I and III**,** mainly generated ETC to supply ATP for cells, which made electrons leak to generate ROS superoxide. Sustained elevated ROS levels could cause detrimental effects (Quinlan et al., 2013; Shadel and Horvath, 2015). Mitochondrial quality is often assessed by mitochondrial fission and fusion which is an essential signal in cell signal transduction (Chandel, 2015; Idelchik et al., 2017). These results identified that nebivolol disturbed mitochondrial function in OSCC cells.

**FIGURE 7:**
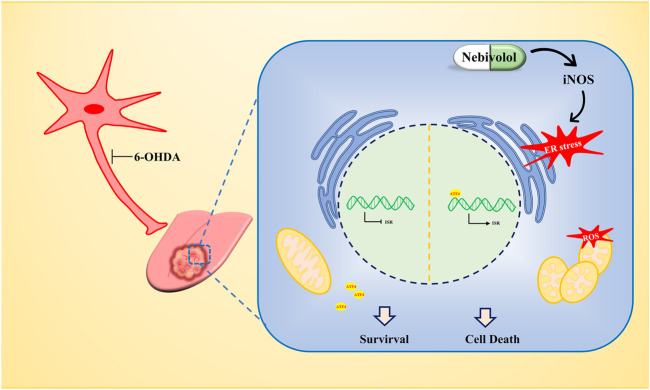
The antitumor mechanism of nebivolol in OSCC cells. Nebivolol triggered the accumulation of iNOS to activate ER stress. Meanwhile, mitochondrial dysfunction was stimulated to enhance ROS level to induce apoptosis in nebivolol-treated OSCC cells.

In summary, the TME fulfills these functions by secreting molecules to affect and regulate its phenotype which became the basis for enhancing cancer progression. Our findings demonstrated that nerves infiltrated in the TME which could be regarded as drug targets to affect the cancer progression. Although chemical denervation by using 6OHDA was successfully applied in mice models of OSCC treatment, the cytotoxic effects limited its clinical application. Adrenergic blockade by nebivolol administration showed great potential to be implemented in humans. Mechanistically, nebivolol could promote iNOS expression and NO accumulation, which resulted in activation of the ER stress. ER stress arrested OSCC cell growth and simultaneously induced mitochondrial dysfunction. Due to impaired ETC, nebivolol increased the OXPHOS protein expression but dramatically decreased the respiration capability. The malfunctioning mitochondria enhanced the production of ROS and led to OSCC cell death. Therefore, repurposing nebivolol, as targeting adrenergic nerve fibers, was verified as an effective strategy to halt the progression of OSCC (Figure 7).

## Conclusion

Our preclinical data demonstrated that nebivolol halted oral mucosa carcinogenesis. Repurposing nebivolol to treat OSCC could be represented as an effective strategy. Remarkably, the action of nebivolol was mediated by activation of ER stress *via* increased iNOS which subsequently triggered dysfunctional mitochondria. The disordered mitochondria function caused the accumulation of ROS, resulting in an antitumor effect on OSCC cells. Nebivolol-induced apoptosis may provide new strategies for treating OSCC cells.

## Data Availability

The raw data supporting the conclusions of this article will be made available by the authors, without undue reservation.
