# Supplementary material for: Adrenergic Blockade by Nebivolol to Suppress Oral Squamous Cell Carcinoma Growth via Endoplasmic Reticulum Stress and Mitochondria Dysfunction
Source: Front Pharmacol. 2021 Aug 12;12:691998. doi: 10.3389/fphar.2021.691998 (PMC8387679; doi:10.3389/fphar.2021.691998)
Supplement: Supplementary file 1 [file Table1.DOCX]

| **Genes** | **Forward primer sequences** | **Reverse primer sequences** |
| --- | --- | --- |
| ATF4 | ATGGATTTGAAGGAGTTCGACT | AGAGATCACAAGTGTCATCCAA |
| Cdsn | CACCTTCTCAGACCCTTGTAAG | GCTTAAAAGATCCTGCAGAACC |
| Trib3 | CTACGTGGGACCTGAGATACTC | GAGTCCTGGAAGGGGTAGT |
| Gadd45α | CAGATCCACTTCACCCTGATC | GATGAATGTGGATTCGTCACCA |
| ClpP | CCTATGACATCTACTCGCGG | CTTCTTGTTGCTCTCGGATTG |
| ATF3 | TAGCCCCTGAAGAAGATGAAAG | CTTCTTCTTGTTTCGGCACTTT |
| CHOP | GAGAATGAAAGGAAAGTGGCAC | ATTCACCATTCGGTCAATCAGA |
| Fgf21 | GAAGCCGGGAGTTATTCAAATC | ACATTGTATCCGTCCTCAAGAA |
| Gdf15 | CTGGTGTTGCTGGTGCTCTCG | TCGGAATCTGGAGTCTTCGGAGTG |
| iNOS | GACTTTCCAAGACACACTTCAC | TTCGATAGCTTGAGGTAGAAGC |
| eNOS | GATGTTACCATGGCAACCAAC | GAAAATGTCTTCGTGGTAGCG |
| nNOS | CTTAAGAGCACATTGGAAACGG | CAATAAACTCTTTGGCGAGAGG |
| GAPDH | CCTGTTCGATCAGCCG | CGACCAAATCCGTTGACTCC |
