# Supplementary figures and images for: Adrenergic Blockade by Nebivolol to Suppress Oral Squamous Cell Carcinoma Growth via Endoplasmic Reticulum Stress and Mitochondria Dysfunction
Source: Front Pharmacol. 2021 Aug 12;12:691998. doi: 10.3389/fphar.2021.691998 (PMC8387679; doi:10.3389/fphar.2021.691998)

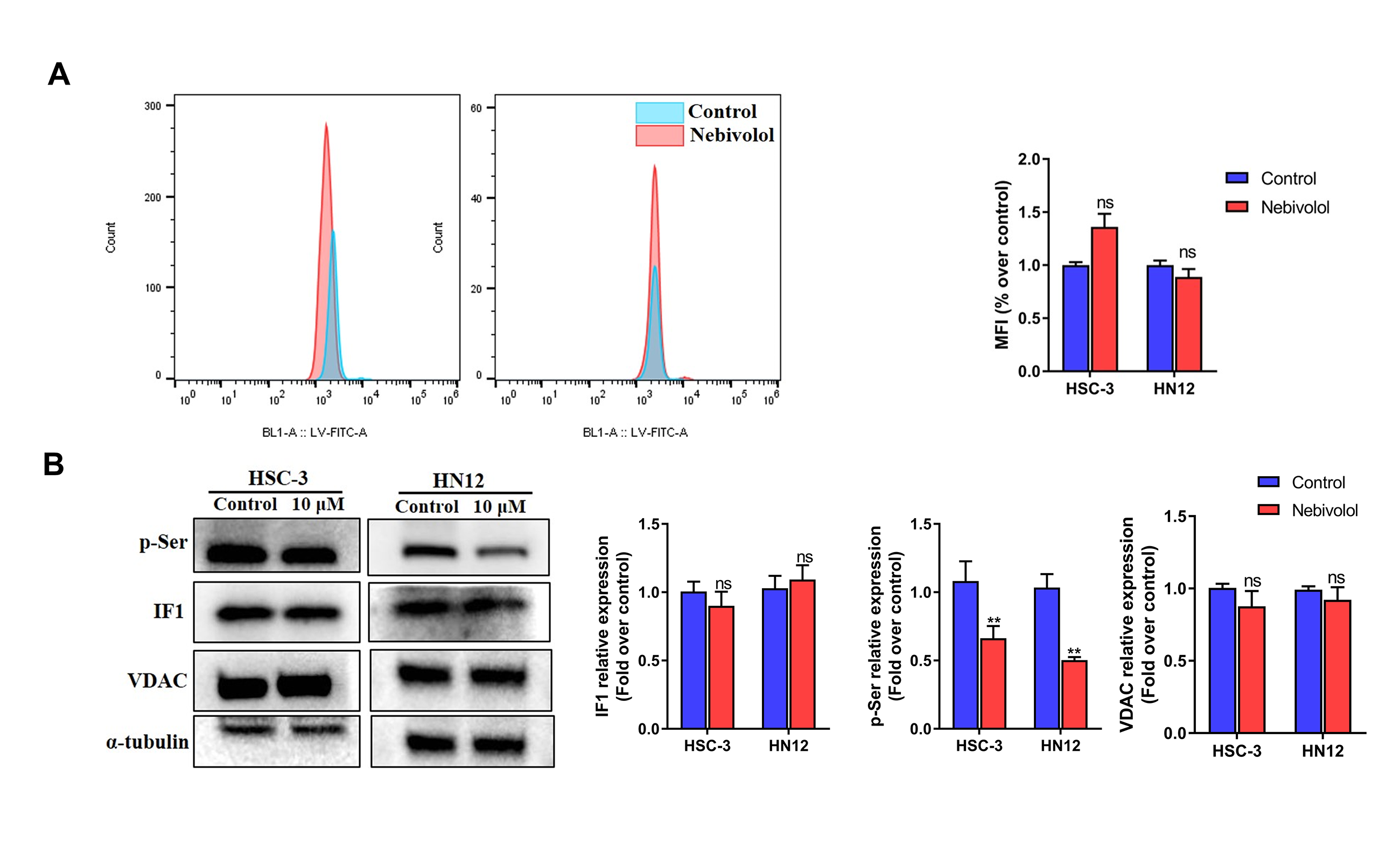

Supplement: Supplementary file 2 [file Image2.TIF]

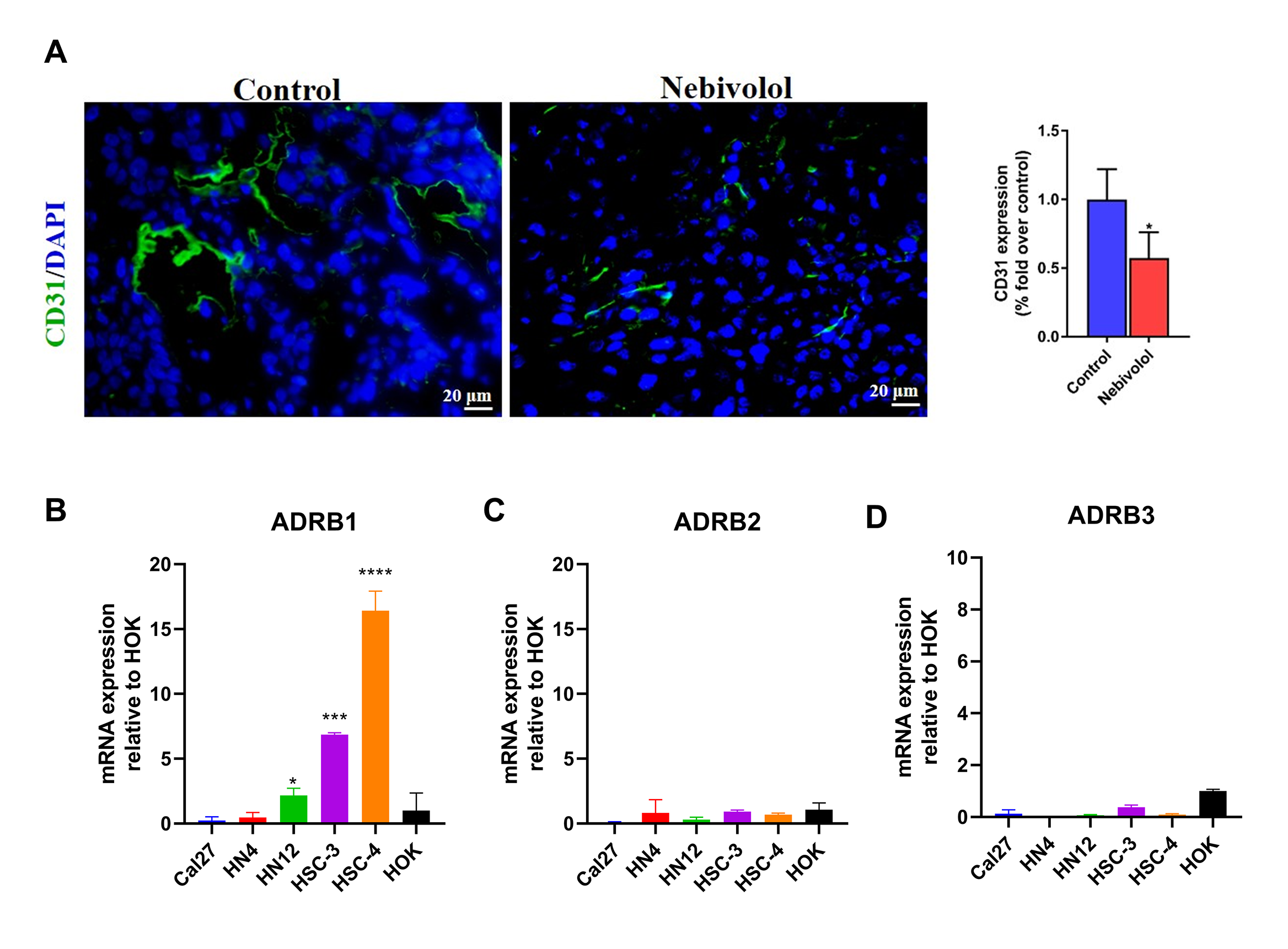

Supplement: Supplementary file 3 [file Image1.TIF]
